# Natural products modulating MAPK for CRC treatment: a promising strategy

**DOI:** 10.3389/fphar.2025.1514486

**Published:** 2025-03-05

**Authors:** Lin Zhou, Jinlong Zhang, Kangning Zhao, Bo Chen, Zhen Sun

**Affiliations:** ^1^ The First Clinical Medical College, Shandong University of traditional Chinese medicine, Jinan, China; ^2^ Department of Gastroenterology, Nanjing Hospital of Chinese Medicine Affiliated to Nanjing University of Chinese Medicine, Nanjing, China; ^3^ The Second Gastroenterology Department, Affiliated Hospital of Shandong University of Traditional Chinese Medicine, Jinan, China

**Keywords:** colorectal cancer, natural products, MAPK pathway, ERK, p38, JNK

## Abstract

Colorectal cancer (CRC) is a common malignant tumor of the digestive system, and the pathogenic mechanism is still unclear, mostly related to genetics, immunity, inflammation, and abnormal activation of tumor-related signaling pathways. MAPK belongs to the Ser/Thr kinase family, which plays an important role in complex cellular programs such as the regulation of cell proliferation, differentiation, apoptosis, angiogenesis, and tumor metastasis. Increasing evidence supports that MAPK activation is highly correlated with the risk of CRC. Targeting MAPK may be a therapeutic strategy, and natural products show great therapeutic potential in regulating MAPK-related proteins. In this paper, we searched PubMed, Web of Science and CNKI databases with keywords “colorectal cancer, natural products, MAPK pathway, ERK, P38, JNK” for relevant studies in the last 14 years from 2010 to 2024. This work retrieved 47 studies, aiming to provide new therapeutic strategies for CRC patients and lay the foundation for new drug development.

## 1 Introduction

Colorectal cancer (CRC) is the third most commonly diagnosed cancer and the second leading cause of cancer deaths globally in 2022, and the second and third most common cancers in women and men respectively (Siegel et al., 2024). Currently, clinical treatments for colorectal cancer include surgery, radiotherapy and chemotherapy, but all of them have poor prognosis and drug resistance, which seriously reduce the survival and quality of life of CRC patients. Therefore, seeking new therapeutic strategies has become an urgent problem nowadays. In recent years, botanical drugs have shown strong therapeutic potential in the treatment of oncologic diseases. With the characteristics of multi-targets, low side effects and good efficacy, botanical drugs can inhibit tumor development by inhibiting the EMT process, blocking angiogenesis, regulating apoptosis and autophagy, promoting cell cycle arrest, and inhibiting the proliferation, invasion and migration of cancer cells, and it has been widely applied to a variety of oncological diseases, such as gastric cancer, hepatocellular carcinoma, and breast cancer (Wang K. et al., 2021). Mitogen-activated protein kinase (MAPK) is a serine/threonine-specific protein kinase, and activated MAPK is involved in the regulation of cell proliferation, differentiation, apoptosis, angiogenesis and tumor metastasis (Widmann et al., 1999).

Astragaloside IV (AS-IV), a triterpenoid extracted from botanical drug *Astragalus mongholicus* Bunge, has anti-inflammatory, anti-oxidative stress, and anti-tumor effects. Jiang et al. (2017) found that AS-IV inhibited the activation of MAPK in MDA-MB-231 breast cancer cells, thereby inhibiting cell proliferation and invasion. In addition, Holothurin A (HA), a triterpenoid saponin extracted from Holothuria scabra, inhibited P38 MAPK and Akt/JNK and blocked the EMT process in PC3 cells of prostate cancer by upregulating the expression of E-calmodulin and downregulating the expression of waviness proteins, twist1, slug and snail (Janta et al., 2023).

Aberrant activation of the MAPK pathway is closely associated with the development of many cancers, and studies suggest that natural products can intervene in MAPK, which is a potent therapeutic strategy for tumor treatment. Notably, the MAPK is also involved in chemotherapy resistance in CRC cells, and the sensitivity of CRC cells to drugs can be enhanced by modulating MAPK. In addition, inhibiting the activation of p38δ MAPK suppresses the growth of CRC cells, improves the efficacy of 5-FU, and overcomes drug resistance to some extent (Stramucci and Bossi, 2019).

Plenty of natural products are candidates for the treatment of CRC, and these metabolites have been shown to act by modulating MAPK-related protein molecules. Thus, these natural products could be complementary and alternative treatments for CRC.

## 2 Overview of the MAPK signaling pathway

**FIGURE 1:**
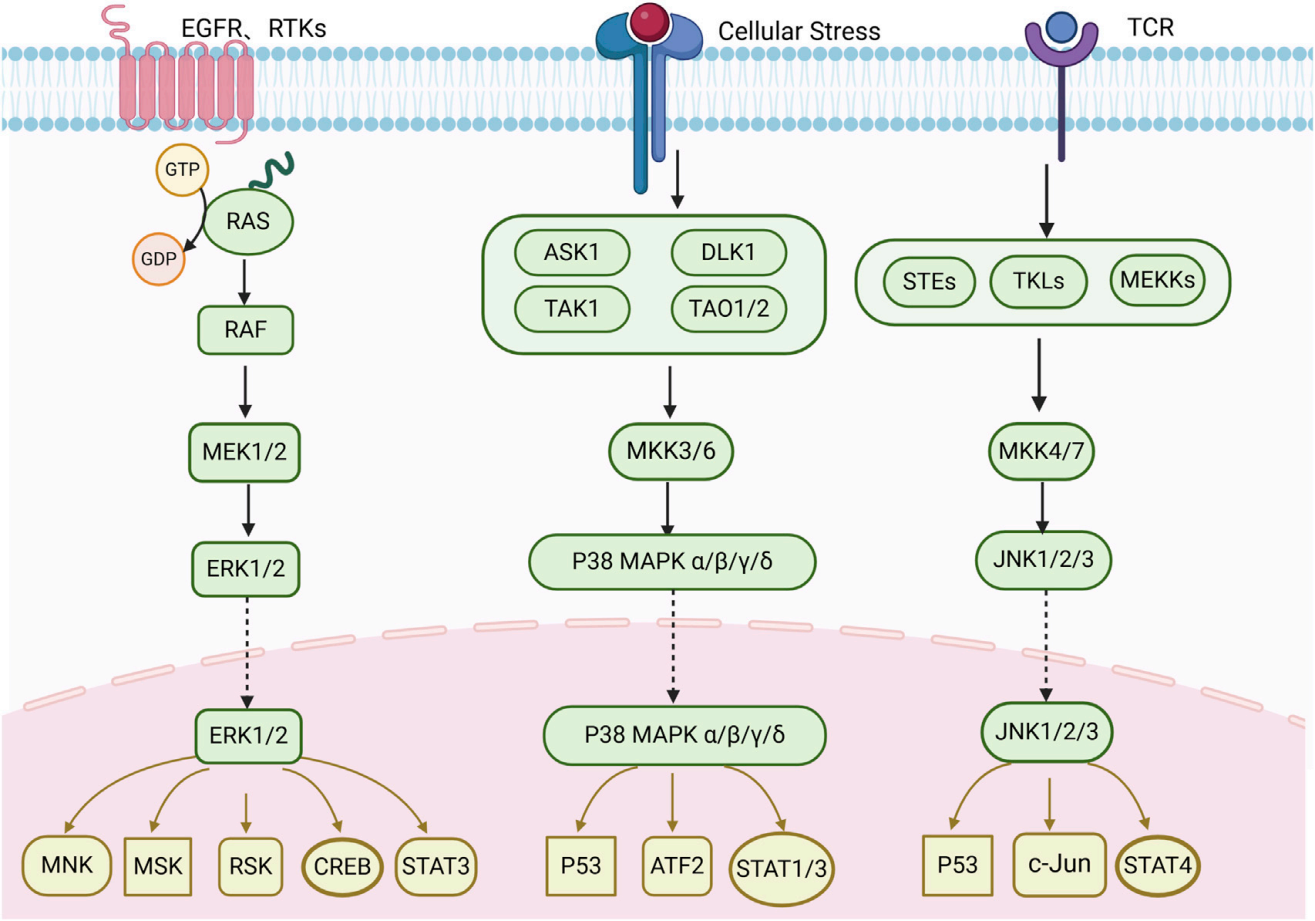
Mechanism of MAPK. Created with Biorender.com.

The MAPK pathway is involved in the regulation of a variety of cellular physiological and pathological processes, including cell proliferation, differentiation, and apoptosis. This signaling pathway also plays an important role in a variety of diseases, such as tumorigenesis, cardiovascular diseases, and immune cell activation (Lee et al., 2020). The MAPK family involved in mammals mainly includes extracellular regulating kinase (ERK), p38 mitogen-activated protein kinase (p38 MAPK/p38) and c-Jun N-terminal kinase (JNK) signaling pathways (Fang and Richardson, 2005) (Figure 1).

### 2.1 ERK1/2

The ERK (classical MAPK pathway) is crucial for cells to respond to external stimuli like growth factors. It activates cell cycle and anti-apoptotic proteins, driving cell-cycle progression and escaping programmed cell death (Martin-Vega and Cobb, 2023). Ras small GTPase (RAS) is activated by GTP/GDP exchange factors (GEFs) in response to stimuli such as receptor tyrosine kinases (RTKs) and EGFR (epidermal factor receptor). Activated RAS recruits RAF to the cell membrane and activates RAF. Then, activated RAF phosphorylates and activates MEK1/2 (Song et al., 2022a). MEK1/2 phosphorylate ERK1 (T202/Y204) and ERK2 (T183/Y185). Phosphorylated ERK translocates to the nucleus, activating transcription factors that regulate cell processes like proliferation, survival, growth, metabolism, migration, and differentiation (Ullah et al., 2022). KRAS and BRAF mutations are common in cancer cells. Aberrant ERK activation promotes tumor growth, survival, invasion, metastasis, extracellular matrix degradation, and angiogenesis (Asl et al., 2021). Zhao et al. (2017) found that increased ERK expression can promote migration and invasion. Jenkins et al. (2023) showed that MAPK/ERK inhibitors can suppress proliferation and angiogenesis.

### 2.2 p38 MAPK

The p38MAPK cascade, also called the p38 pathway, is involved in cellular stress, immune response and inflammation. It has four isoforms: p38α (MAPK14), p38β (MAPK11), p38γ (MAPK12) and p38δ (MAPK13) (Han et al., 2020). A variety of stimuli such as oxidative stress, genotoxic and DNA damaging agents, inflammatory cytokines, PAMPs (Pathogen-Associated Molecular Patterns) and DAMPs (Danger-Associated Molecular Patterns) can activate p38 (Kyriakis and Avruch, 2012). Signals are transmitted via a complex network of signaling molecules, often activating small GTPases (Rac and CDC42), sometimes through activating interactions of adaptor proteins, and then activating protein kinases in the MAP4K or direct MAP3K layer of the p38 cascade (Sanz-Ezquerro and Cuenda, 2021). Activated p38 accumulates transiently and moves to the nucleus, activating downstream signaling proteins that regulate processes like RNA splicing, cytokine production, inflammatory responses, apoptosis, autophagy and cell cycle arrest (Wang et al., 2019). Kassassir et al. (2023) found that inhibiting p38 phosphorylation reduced the proliferation and invasion of CRC cells. Huang et al. (2023) reported that inhibiting IFI6 reversed OXA resistance in CRC cells by promoting ROS-induced p38. These studies suggest that regulating the p38 pathway may be a new approach for developing drugs to treat CRC in the future.

### 2.3 JNK

The JNK pathway (c-Jun N-terminal Kinases), also called the stress-activated protein kinase pathway, is crucial in cellular stress responses like regulating cell death and inflammatory responses (Wu et al., 2019). Encoded by JNK1, JNK2, and JNK3 genes, it's mainly activated by MKK4 andMKK7. When cells are stressed by irritants, growth factors or cytokines, the JNK pathway is activated. The activation signal passes through the Ras superfamily of G proteins to MLK, then activates MKK4/7 and downstream JNK (Tam and Law, 2021). The JNK pathway is important for apoptosis, inflammation, cytokine production and metabolism. Li et al. (2021a) showed that silencing JNK can inhibit CRC cell expansion. Alotaibi AG et al. (2021) also found that inhibiting JNK could reduce TNF-a-induced DNA damage and improve the RC tumor microenvironment.

## 3 Mechanism of MAPK in CRC

### 3.1 MAPK is involved in the regulation of cellular activity

MAPK pathway significantly regulates CRC cell growth, proliferation, migration, and invasion. Xu G. et al. (2023) discovered that CEMIP, a cell migration-inducing protein, activated the CDC42/MAPK signaling pathway by enhancing the ubiquitination and degradation of the tumor suppressor GRAF1, promoting the shift from an epithelial to a mesenchymal phenotype, and ultimately facilitating the migration and invasion of HCT116 and SW480 cells. Li et al. (2021b) found that in CRC cells, the transcription factor RBP-J was highly expressed compared to normal tissues which elevated the expression of Tiam1, phosphorylates p38 MAPK, Slug-1, Twist1, and MMP-9, promoting the proliferation, migration, and invasion. Jin Y. et al. (2022) reported that increasing the level of p-JNK activated the MAPK downstream transcription factor ATF2, which then activated UCA1 to promote the migration and invasion of CRC cells. Collectively, these studies indicate that activated MAPK promotes CRC cell growth, proliferation, migration, and invasion through multiple mechanisms which include facilitating the epithelial-mesenchymal transition, decreasing the levels of proteins like Slug, waveform, increasing Slug-1, and Twist1, and inducing the activation of relevant transcription factors.

### 3.2 MAPK mediates autophagy and apoptosis in CRC

Cellular autophagy is an intracellular self-degradation and recycling process vital for maintaining intracellular homeostasis, removing damaged metabolites, responding to stress, and regulating cell growth and survival. It occurs in four stages: induction/activation, nucleation/extension, autophagosome-lysosome fusion, and content degradation. Studies indicated that autophagy play a significant role in cancer development (Debnath et al., 2023). P38 MAPK is an important autophagy-related protein, which activated via TAK1-MKK3/6-p38 and ASK1-MKK3/6-p38. Activated p38 phosphorylates Atg5 at T75, inhibiting autophagic membrane elongation and the LCI to LCII conversion, thus suppressing the autophagy pathway (Keil et al., 2013). Regulating p38 expression can inhibit colorectal cancer cell growth and offer an effective CRC treatment method. Zheng et al. (2020) demonstrated that in HCT116 and HT29 cells, inhibiting PRDX2 reduced p38 phosphorylation, lowered the LC3II/I ratio, increased P62 expression, and decreased Beclin 1 expression, disrupting autophagy’s protective effect and increasing apoptosis. Conversely, Stramucci et al. (2019) found that silencing MKK3 decreased p38δ MAPK phosphorylation, reduced the late autophagosome marker p62/SQSTM1 levels, and increased the LC3II/LC3I ratio, inducing cellular autophagy and inhibiting tumor survival in HT29 and HCT-116 cells.

Apoptosis is a precisely regulated programmed cell death, occurring in development and aging, and maintaining cell populations in tissues. It has two main caspase-mediated pathways: the intrinsic pathway, releasing cytochrome c from mitochondria and controlled by bcl-2 family proteins (anti-apoptotic bcl-2 and pro-apoptotic bax and bak), and the extrinsic pathway activated by death receptors like Fas and other tumor necrosis factor receptor family members. The MAPK signaling pathway is crucial in apoptosis regulation. Eukaryotic elongation factor 1 A1 (eEF1A1) overexpression promotes CRC proliferation. Fan et al. (2023) found that silencing eEF1A1 in RKO and Caco2 cells was able to downregulate ERK, p38, JNK phosphorylation, to reduce PCNA expression, to upregulate cleaved-caspase 3, Bax an to downregulate Bcl-2, inducing apoptosis and inhibiting cell proliferation. MicroRNA-125, a highly conserved miRNA, could target and downregulate VEGF, inhibit the downstream MAPK signaling pathway (decreasing ERK, p38, and JNK phosphorylation), enhance caspase-3 activity, increase DNA fragmentation, inducing apoptosis in RKO cells (Wu et al., 2018). The above studies show that during CRC formation, the balance between cell growth and apoptosis is disrupted. MAPK activation regulates multiple transcription factors, increasing pro-apoptotic protein expression and decreasing anti-apoptotic protein expression, thus inducing apoptosis in CRC cells.

### 3.3 MAPK and oxidative stress

Oxidative stress occurs when there’s an imbalance between reactive oxygen species (ROS) and antioxidants. ROS play roles in cell processes like proliferation, differentiation, migration, apoptosis, and necrosis (Ebrahimi et al., 2020). Excessive formation of ROS leads to a disruption of redox homeostasis, which in turn leads to oxidative stress, often regulating the MAPK signaling pathway. Oxidative stress can activate JNK and p38 MAPK, inducing the co-stimulatory molecule CD80, activating the transcription factor STAT3, and influencing CRC development in terms of immune defense (Marchiori et al., 2019). MLK3, a MAP3K, has kinase-independent activation. Schroyer et al. (2018) found that oxidative stress activates ERK1/2, phosphorylates MLK3 at Ser705 and Ser758, promotes its interaction with B-Raf to activate ERK1/2, forming the ERK1/2-MLK3-B-Raf-MEK1/2-ERK1/2 mechanism, which promotes H_2_O_2_-treated HCT116 cell invasion. Jiang et al. (2023) discovered that in HCT116 cells, p20BAP31 was highly upregulated, which upregulated ROS levels and activated JNK phosphorylation, leading to mitochondria-dependent apoptosis and S-phase cell cycle arrest. Meng et al. (2018) cultured SW480 cells with quinuclidin (1,2,5,8-tetrahydroxyanthraquinone), and found it was able to produce ROS in a time-dependent manner, to increased JNK and p38 phosphorylation, to decreased ERK phosphorylation, inducing caspase-3-dependent apoptosis and causing G2/M-phase cell cycle arrest by activating p53, upregulating Bad, downregulating Bcl-2. ROS scavenger NAC inhibits these processes. Overall, oxidative stress-induced ROS affect cell processes and CRC-related events through MAPK and other molecular mechanisms.

### 3.4 MAPK and pathologic angiogenesis

Angiogenesis is the formation of new blood vessels from existing capillaries, regulated by pro -and anti-angiogenic factors (Lugano et al., 2020). Malignant tumors rely on angiogenesis due to enormous demand for nutrients, oxygen and natural aggressiveness. The MAPK pathway is involved in angiogenesis regulation. ELK4, a MAPK downstream target, regulates the transcription factors SP1 and SP3, activates the transcription of pathological neoangiogenic factor LRG1, and promotes tumor angiogenesis, cell proliferation, and migration in HCT116 and LoVo cells (Zhu et al., 2023a). Klemke et al. (2021) found that macrophage migration inhibitory factor (MIF) promotes angiogenesis and cell proliferation in HCT116 cells by activating the CD74 receptor, increasing phosphorylation of p38 and ERK, and upregulating the expression of VEGF and IL8.In an AOM/DSS-constructed CRC mouse model, the p38MAPK pathway was activated and its key effector kinase MK2 increased the expression of pro-angiogenic factors Serpin-E1 and Cxc-12, promoting tumor angiogenesis and metastasis (Suarez-Lopez et al., 2021). Elevated MicroRNA 452 in Caco2 cells, could significantly decrease VEGFA protein levels and inhibit the KRAS-BRAF-MAPK pathway. *In vitro* experiment also demonstrated that MicroRNA 452 could decrease the expression of angiogenic marker CD31 and inhibit angiogenesis (Mo et al., 2019). Upregulated ILT4 (Immunoglobulin-like transcript 4) in SW620 and HCT116 cells was able to activate the ERK pathway and upregulate the levels of VEGF-A and FGF-1, promoting angiogenesis, while downregulating ILT4 enhances bevacizumab’s anti-vascular and anti-tumor ability (Liu J. et al., 2023). In summary, multiple factors and molecules, including those related to the MAPK pathway, either promote or inhibit angiogenesis in CRC cells and mouse models through different mechanisms.

## 4 Natural products inhibit CRC by regulating MAPK

Numerous experimental findings have demonstrated that natural products can exert beneficial effects on colorectal cancer (CRC) treatment. By modulating the MAPK signaling pathway, they can enhance the state of pathological tissues, suppress the proliferation and migration capabilities of tumor cells, and reduce drug resistance. These properties indicate natural products with a significant role in CRC treatment, thus providing a reliable theoretical foundation for the utilization of natural products in treating CRC. In this review, a total of 60 articles were retrieved. After excluding 13 articles, 47 remained. The majority of these were cellular experiments, with 10 involving animal experiments. The review covered 10 types of flavonoids, 11 types of terpenes, 7 types of saponins, 2 types of polyphenols, 5 types of alkaloids, and 12 types of other metabolites, as shown in Table 1.

**TABLE 1:** Natural products against colorectal cancer by modulating MAPK.

Extract	Origination	Structure	Model	Biological effects	Results	References
Licochalcone B	*Glycyrrhiza glabra* L	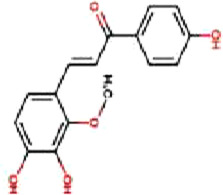	HCT116-OxR cells	JNK↑ p38 MAPK↑ p21↑ p27↑ B1↓ cdc2↓	G2/M cell cycle arrest Inhibit proliferation Induce apoptosis	Kwak et al. (2023a)
Luteolin	*Lonicera japonica* Thunb	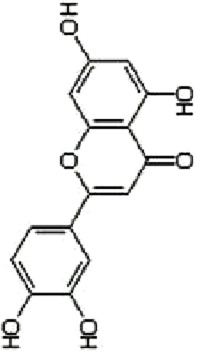	HCT-116 cells HT-29 cells BALB /c nude mice	p-MEK1↓ p-ERK1/2↓ BCL-2↓ BAX↑ cleaved caspase-3↑	DNA damage Induce apoptosis G2 cell cycle arrest Reduce tumor weight and volume	Song et al. (2022a)
			HT-29 cells	JNK↑ p38↑ ROS↓ SOD↑ CAT↑	Induces apoptosis	Kang et al. (2017)
Kaempferol	*Kaempferia galanga* L	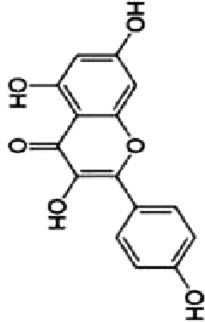	HCT116 cells HCT15 cells	ROS↑ p38 MAPK↑ caspase-8↑ -9↑ -3↑ cleaved PARP↑	Induce apoptosis	Choi et al. (2018)
			5-FU-resistant LS174-R cells	P38↑ P21↑ p53↑ Ser15↑ p-cdc2↓ p27↓	Induce apoptosis Inhibit tumor growth	Riahi-Chebbi et al. (2019)
Quercetin	*Agrimonia pilosa* Ledeb	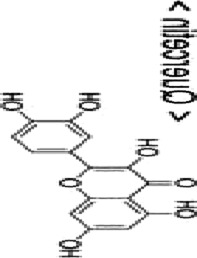	RKO cells SW480 cells HCT116 cells Balb/c nude mice	JNK↑ E-calmodulin↑ waveform protein↓ N-calmodulin↓	Inhibit invasion Inhibit migration Inhibit Tumor growth	Trinh et al. (2022)
Bavachin	*Cullen corylifolium* Medik	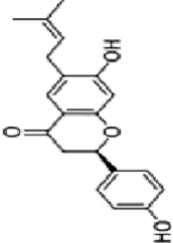	HT29 celld HCT 116 cells Balb/c nude mice	p38↑ ERK↑ JNK↑ Gadd45a↑ PARP↑ cleaved Caspase-3↑	Induce apoptosis Inhibit Tumor growth	Wang et al. (2023)
Apigenin	*Apium graveolens* L	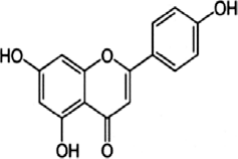	SW480 cells HCT-116 cells	P38 MAPK ↓ Bax↑ cleaved caspase-3↑ Bcl-2↓ MMP2↓ MMP9↓ Snail↓ E-calmodulin↑	Induce apoptosis Inhibit cell growth and metastasis	Zhang et al. (2021)
Chrysin	*Oroxylum indicum* L	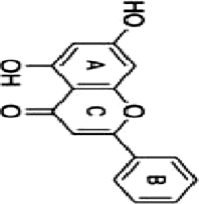				
Echinatin	*Trigonella foenum-graecum* L	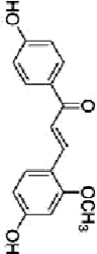	HT116 cells HT29 cells HCT116-OxR cells HT29-OxR cells	ROS↑ GRP78↑ CHOP↑ p-JNK↑ p-p38↑	G2/M cell cycle arrest Induce apoptosis	Kwak et al. (2023b)
Baicalein	*Scutellaria baicalensis* Georgi	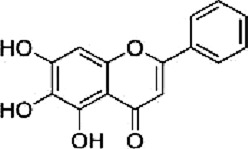	HCT 116 cells	JNK↑ ERK↑ p38↑ DEPP↑ Gadd45a↑ Cleaved caspase-3↑ -9↑	Induce apoptosis	Su et al. (2018)
Origanum majoana Essential Oil	*Origanum vulgare* L	------	HT-29 cells	P38 MAPK↑ Cleaved p70S6K↑ caspase-3↑ TNF-α↑	Induce apoptosis	Athamneh et al. (2020)
Tanshinone I	*Salvia miltiorrhiza* Bunge	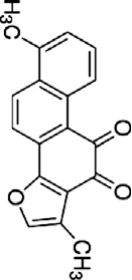	HCT116 cells HT29 cells	Cleaved PARP↑ tBid↑ caspase-3↑ ‐8↑ p38↑ JNK↑	Induce apoptosis	Kim et al. (2018)
Oridonin	*Isodon rubescens* (Hemsl.)	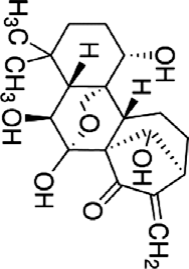	HCT116 cells	p-p38↑ BMP7↑ Bad↑ Bcl-2↓	G2 cell cycle arrest Inhibit proliferation Induce apoptosis	Ren et al. (2016)
Cryptotanshinone	*Salvia miltiorrhiza* Bunge	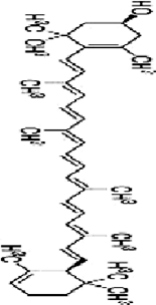	SW620-Ad300 cells	LC3-II↑ ROS↑ P38 MAPK↑ NF-kB↑	Induce autophagy	Xu et al. (2017)
Andrographolide	*Andrographis paniculata* (Burm.f.) Wall. ex Nees	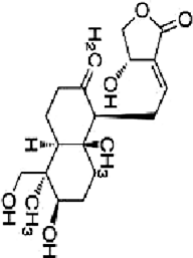	HCT116 cells	NADPH↓ ERK1/2↓ P38 MAPK↓ IL-8↓	Inhibit angiogenesis	Yuan et al. (2018)
Alisol B 23-acetate	*Alisma plantago-aquatica subsp*. orientale (Sam.) Sam	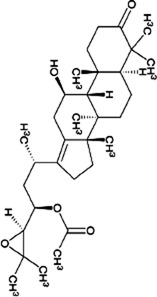	C57BL/6J mice	p38-MAPK↓ERK↓ JNK↓ IL-6↓ TNF-α↓ IFN-γ↓ IL-10↑	Prevention of CAC	Zhu et al. (2021)
TS essential oil	*Thymus algeriensis* Boiss. & Reut	-------	HCT116 cells	ERK1/2↑ JNK↑ p38↑ DR5↑ SP1↑ CHOP↑	Induce apoptosis	Guesmi et al. (2021)
Ganoderma lucidum triterpenes	*Ganoderma lucidum* (Curtis) P. Karst	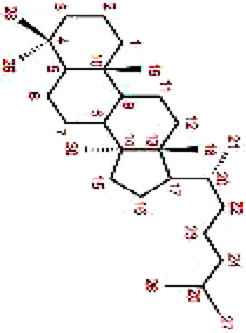	HT-29 cells	p-p38↓ Beclin-1↑ LC-3↑	Induce autophagy	Thyagarajan et al. (2010)
Yuanhuacine	*Daphne genkwa Siebold & Zucc*	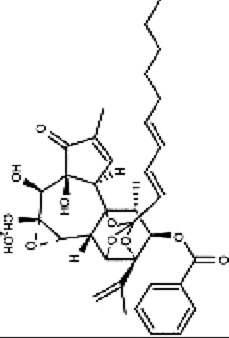	HCT116 cells	p38 MAPK↑ p21 mRNA↑	Induced cell arrest in G2/M phase	Zhang et al. (2014)
Crocetin	*Crocus sativus* L	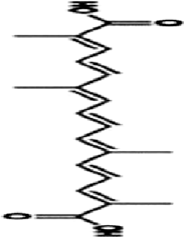	HCT-116 cells	p-p38 MAPK↑ VEGF↓ MMP-9 mRNA↓	Inhibit proliferation Inhibit invasion Inhibit angiogenesis	Khajeh et al. (2020)
Deguelin	*Mundulea sericea* (Willd.) A.Chev	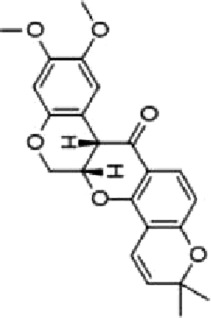	SW620 cells RKO cells	p38 MAPK↑ cleaved caspase-3↑ cleaved PARP↑ Bcl-2↓	Inhibit proliferation Induce apoptosis	Chen et al. (2018)
Ginsenoside Rh1	*Panax ginseng* C. A. Mey.	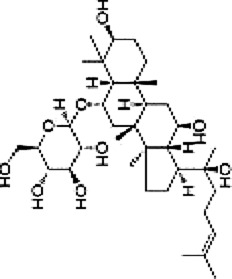	SW620 cells BALB/c nude mice	p-P38/P38↓ p-ERK1/2/ERK1-2 ↓ p-JNK/JNK↓ MMP1↓ MMP3↓ TIMP3↑	Inhibit invasion and migration Inhibit Tumor growth	Lyu et al. (2019)
Rhizoma Panacis Majoris	*Panax bipinnatifidus var*. bipinnatifidus	--------	HCT116 cells SW620 cells	p-JNK/JNK↑ p-p38/p38↑ p-ERK/ERK↓ Bcl-2↓ Bcl-xL↓ Mcl-1↓	Induced apoptosis Blocked cell cycle in G0/G1 and S phase	Chang et al. (2021)
Dioscin	*Dioscorea panthaica* Prain & Burkill	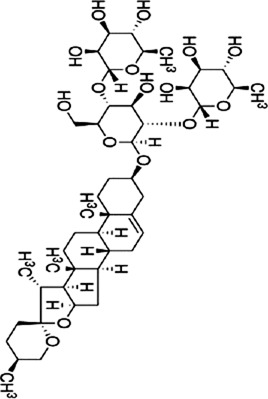	RKO cells HT-29 cells	JNK↑ p38 MAPK↑ caspase-3↑ -9↑ cleaved PARP↑	Induce apoptosis	Li et al. (2014)
			C26 derived tumor mouse	VEGF↓ p-ERK1/2↓	Inhibit angiogenesis	Tong et al. (2014)
Platycodin D	*Platycodon grandiflorus* (Jacq.) A.DC	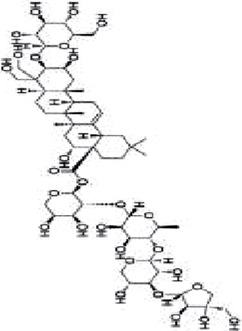	HT-29 cells	p-p38↑ Bax↑ Cleaved PARP↑ Bcl-2↓	Induce apoptosis	Han et al. (2024)
Paris saponin VII	*Trillium tschonoskii* Maxim	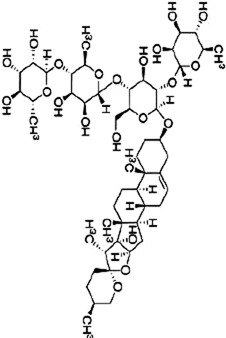	HT-29 cells SW-620 cells	D1↓ Cdk-4↓ Cdk-6↓ ERK1/2↓ Cleaved caspase-3↑ -9↑ PARP↑ Bax↑ Bcl-2↓	G1 cell cycle arrest Induce apoptosis	Li et al. (2014)
Gynostemma pentaphyllum saponins	*Gynostemma pentaphyllum* (Thunb.) Makino	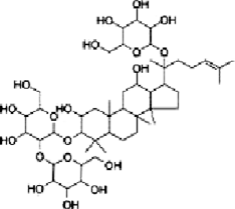	ApcMin/+ CRC mice	Prdx1↑Prdx2↑ Raf-1↓ RAS↓ RAF↓ MEK↓ ERK↓	Reduce the number and size of intestinal polyps	Tai et al. (2016)
Curcumin	*Curcuma longa* L	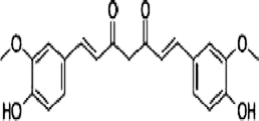	CT26 cells	P38 MAPK↓ HPSE↓	Inhibit invasion Inhibit migration	Li et al. (2020)
[6]-Gingerol	*Zingiber officinale* Roscoe	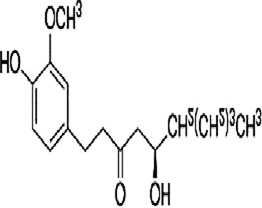	SW-480 cells	ERK1/2↓ JNK↓ Caspase -3↑ -7↑ Cleaved PARP↑	Induce apoptosis	Radhakrishnan et al. (2014)
Lycorine	*Lycoris radiata* (L'Hér.) Herb	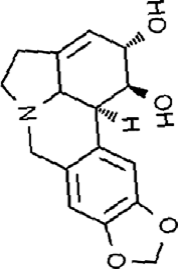	CT-26 cells HCT116 cells HT-29 cells	E-calmodulin↑ N-calmodulin↓ β-linker↓ waveform protein↓ Snai1 protein↓ MAPK↓	Block EMT	Gao et al. (2021)
Piperlongumine	*Piper longum* L	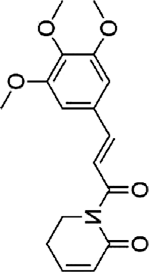	HT-29 cells	ROS↑ ERK↑ caspase-3↑	Induce apoptosis	Randhawa et al. (2013)
Matrine	*Sophora flavescens* Aiton	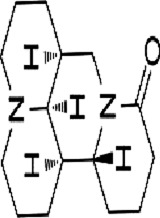	CT26 cells	JNK↓ ERK↓ Cldn9↓ N-calmodulin↓ MMP2↓ MMP9↓	Inhibit proliferation Inhibit invasion Inhibit migration	Du et al. (2023)
Homoharringtonine	*Cephalotaxus hainanensis* H.L.Li	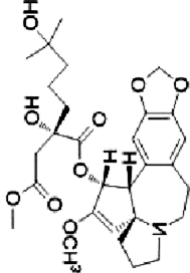	LoVo cells	EphB4↓ ERK1/2↓ Bcl-2↓ Mcl-1↓	Induce apoptosis	Shi et al. (2020)
Tetrandrine	*Stephania tetrandra* S.Moore	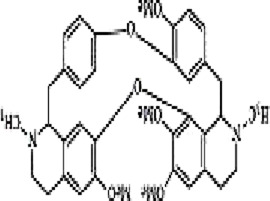	CT-26 cells	P38 MAPK↑	Induce apoptosis	Wu et al. (2010)
Vanillin	*Vanilla pompona* Schiede	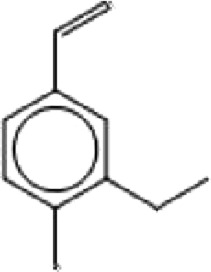	HT-29 cells SW480 cells Male BALB/c nude mice	ASK1-p38 MAPK↑ NNMT↓	Induce apoptosis Inhibit tumor growth	
Podophyllotoxin	*Podophyllum versipelle* Hance	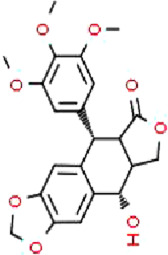	HCT116 cells	ROS↑ RP78↑ CHOP↑ p38 MAPK↑	Oxidative Stress Induce apoptosis G2/M cell cycle arrest	Lee et al. (2021)
Ursolic acid	*Prunella vulgaris* L	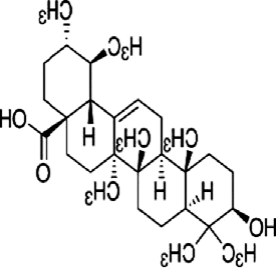	SW620/RKO cells	p-MEK1/2↓ p-ERK1/2↓ p-p38↓ p-JNK↓ caspase-3↑ -9↑ -8↑ Bcl-xL↓ Bcl-2↓ Ki-67↓	Induce apoptosis	Shan et al. (2016)
Di Yu	*Sanguisorba officinalis* L	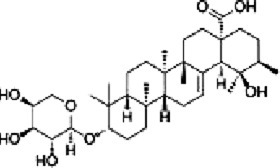	RKO-R cells HCT15-R cells.	Ras/MEK/ERK↓ Bax↑ cleaved caspase-3↑ -9↑ cleaved PARP↑	Enhancing the chemosensitivity to 5-FU Inhibit migration	Zhang et al. (2023)
Ferulic acid	*Angelica sinensis* (Oliv.) Diels	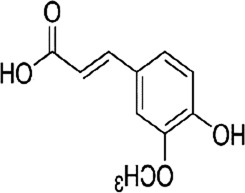	CT26 cells Balb/c mice	JNK↑ ERK↑ BAX↑ LC3-II↑ BCL-2↓	Induce apoptosis Induce autophagy	Chen et al. (2023)
8-Gingerol	*Zingiber officinale* Roscoe	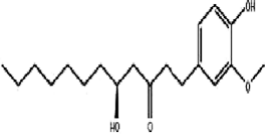	HCT116 cells DLD1 cells	EGFR/STAT/ERK↓ D1↓ c-Myc↓ MMP2↓	Induced cell cycle arrest in the G0/G1 phase Inhibit proliferation Inhibit migration	Hu et al. (2020)
Alantolactone	*Inula helenium* L	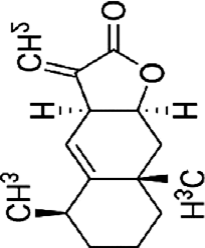	HCT116 cells RKO cells	ROS↑ JNK↑ P38MAPK↑	Inhibit proliferation Induce apoptosis	Cao et al. (2019)
Curcumol	*Curcuma longa* L	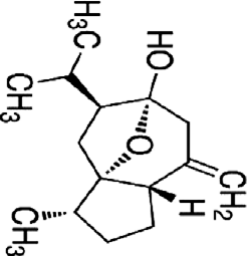	LoVo cells BALB/c athymic nude	IGF-1R↓ p38↑ Bax/Bcl-2↑	Induce apoptosis Inhibit tumor growth	Wang et al. (2015)
Salidroside	*Rhodiola rosea* L	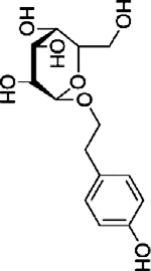	HT29 cells	HT29↑ p-JNK↑ p38 MAPK↑ caspase -3↑ -8↑	Induce apoptosis	El-Kott et al. (2021)
Sulforaphane	*Brassica oleracea* L	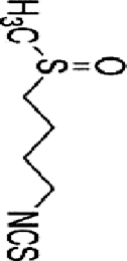	HT-29 cells	ROS↓ MAPK↓ IL-1β↓ IL-6↓	Inhibit proliferation Inhibit migration	Sah et al. (2024)
Imperatorin	*Angelica dahurica* (Hoffm.) Benth. & Hook.f. ex Franch. & Sav	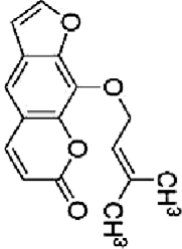	HCT116 cells	ERK1/2↓ JNK↓ p38 MAPK↓ HIF-1α↓ VEGF↓ CD31↓	Inhibit proliferation Inhibit angiogenesis	Mi et al. (2017)
Ganoderma lucidum polysaccharide	*Ganoderma lucidum* (Curtis) P. Karst	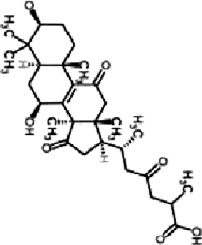	HT-29 cells HCT116 cells	p-ERK↑ LC3-II↓ p62↓	Induce apoptosis	Pan. et al. (2019)

### 4.1 Flavonoids

Flavonoids are important bioactive metabolites that are widely found in a variety of plant medicines. Existing studies on flavonoids mainly focus on antioxidant, anticancer effects (Slika et al., 2022). Some flavonoids, such as licorice chalcone, lignans, kaempferol, and quercetin, have good anti-CRC effects.

Licochalcone B (LCB), a flavonoid extracted from the botanical drugs such as *Glycyrrhiza glabra* L, can treat NLRP3 inflammatory vesicle-associated disorders (Li Q. et al., 2022). Kwak et al. (2023a) cultured oxaliplatin-resistant (OxR) colorectal cancer cells (HCT116-OxR) with different concentrations (10, 20, and 30 µM for 24, 48 h) of LCB. The results showed that LCB significantly upregulated the phosphorylation of JNK and p38 MAPK through ROS-mediated pathways in a dose-dependent manner, and decreased the protein levels of cyclin B1 and cdc2 meanwhile increased the protein levels of p21 and p27, ultimately inducing cell cycle arrest in G2/M phase, apoptosis, which could be reversed by the JNK and p38 Inhibitor SP600125 and SB203580.

Luteolin is a flavonoid extracted from many botanical drugs such as *Lonicera japonica* Thunb, is used in the treatment of colon, lung, and prostate cancers (Çetinkaya and Baran, 2023). Song et al. (2022b) reported that Luteolin (0, 10, 20, and 40 µM) was able to dose-dependently reduce the levels of p-MEK1 and p-ERK1/2, to decrease the expression of BCL-2, to elevated the expression of BAX, cleaved caspase-3, inducing apoptosis and DNA damage, and arresting the cell cycle in G2 phase in HCT-116 and HT-29 cells. *In vivo*, high-dose (40 mg/kg/d) luteolin effectively inhibited tumor growth. Kang et al. (2017) showed that luteolin (5, 10, 25, 50, 200 μg/mL) could dose-dependently increase the phosphorylation of JNK and p38 MAPK, and induce apoptosis through antioxidant effects.

Kaempferol, a major metabolite of the botanical drug *Kaempferia galanga* L, which has antioxidant, anti-inflammatory, and cardioprotective activities (Chen et al., 2024). Riahi-Chebbi et al. (2019) found that 75 µM kaempferol in combination with 60 µM 5-FU was able to induced apoptosis by inhibiting the p38 MAPK pathway and downregulating the expression of phosphorylated-cdc2 and p27 in 5-FU-resistant LS174-R cells. Meanwhile, the combination can reduce the level of VEGF-A and IL-8 and inhibit tumor growth. Choi et al. (2018) reported that kaempferol (0, 12.5, 25, 50 and 100 µM for 24, 48, and 72 h) upregulated the expression of p-p38 MAPK, downregulated the expression of p-JNK and p-ERK, inducing apoptosis in HCT116 and HCT15 cells in a dose- and time manner. The above effects were reversed with the addition of p38 MAPK inhibitor SB203580.

Quercetin is a flavonoid widely found in *Agrimonia pilosa* Ledeb which can treat a variety of cancers such as breast, colorectal, and liver cancer (Maugeri et al., 2023).Trinh et al. (2022) demonstrated that Quercetin (0, 2, 5, 20 and 50 µM for 24,48,72 h) inhibited the invasion,metastasis and effectively induced the MET process in SW480, HCT116 cells by activating JNK, upregulating the expression of E-calmodulin, and downregulating the expression of waveform protein, N-calmodulin. This process can be reversed by JNK inhibitor SP600125. Quercetin could also effectively inhibited the growth of solid tumors in Balb/c nude mice.

Apigenin and chrysin are flavonol flavonoids derived from *Apium graveolens* L and *Oroxylum indicum* L, which have anti-inflammatory and antioxidant properties (Lee et al., 2023; Farkhondeh et al., 2019). Zhang et al. (2021) reported that the combination of apigenin (25 µM) and chrysin (25 µM) inhibited the activity of P38-MAPK/AKT and had a synergistic inhibitory effect on the growth and metastasis of SW480 and HCT-116 cells as compared to the treatment alone. P38 agonist anisomycin can adverse the above process.

Bavachin, a dihydroflavonoid isolated from botanical drugs such as *Cullen corylifolium* Medik, has been used in the treatment of neurological disorders (Wang Y. et al., 2021). Wang et al. (2023) found that Bavachin (20, 30, and 40 μmol·L−1 for 24h, 48 h) increased the phosphorylation level of p38/ERK/JNK in a dose-dependent manner, and upregulated the expression of Gadd45a, cleaved PARP and cleaved Caspase-3, inducing apoptosis and inhibiting tumor growth in HT29 and HCT 116 cells.

Echinatin (Ech), a natural flavonoid extracted from the botanical drug *Trigonella foenum-graecum* L, which has anti-inflammatory properties (Xu et al., 2021). Kwak et al. (2023b) found that Ech (5, 10, and 15 µM) was able to dose-dependently increase ROS levels, to upregulate the expression of GRP78, CHOP, to increase p-JNK and p-p38, inducing apoptosis caused by caspase activation, and blocking the cell cycle at G2/M in oxaliplatin-resistant (HCT116-OxR and HT29-OxR) cells. The inhibitory effects could be abrogated by the JNK inhibitor SP600125 and the p38 inhibitor SB203580.

Baicalein, a flavonoid with anti-inflammatory and anti-tumor effects extracted from the botanical drugs such as *Scutellaria baicalensis* Georgi (Tuli et al., 2020). Su et al. (2018) reported that Baicalein (10, 20, 400 µM for 6, 12, 24 h) was able to dose-dependently upregulate the levels of DEPP, Gadd45a which induced growth arrest and DNA damage, to activate MAPK (JNK/ERK/p38 phosphorylation) which induced apoptosis in HCT116 cells.

The above findings suggest that flavonoids can mediate the MAPK signaling pathway to cause cell cycle arrest, inhibit cell proliferation and induce apoptosis.

### 4.2 Terpenes

Terpenes are a large group of organic metabolites found primarily in leaves, fruits, flowers, rhizomes, and plant roots. Terpenes can be categorized into five main groups: monoterpenes, sesquiterpenes, diterpenes, triterpenes, and heteroterpenes, which have anti-inflammatory, antioxidant, antibacterial, and antitumor properties (Silva-Reis et al., 2023). It has shown significant results in the treatment of CRC.

Origanum majoana Essential Oil (OMEO), a terpenoid metabolite extracted from the botanical drug *Origanum vulgare* L, could be used to treat acne (Taleb et al., 2018). Athamneh et al. (2020) reported that OMEO (256 μg/mL) promoted endogenous and exogenous apoptosis in HT-29 cells by activating p38 MAPK, increasing cleaved p70S6K, and upregulating expression of TNF-α.

Tanshinone I (Tan I), a natural diterpenoid quinone metabolite extracted from botanical drugs such as *Salvia miltiorrhiza* Bunge, has been used to treat with osteosarcoma (Ye et al., 2022). Study (Kim et al., 2018) have shown that Tan I (0, 15, 30, 60, 90, 120 µM for 24 h) dose-dependently activated cleaved PARP, tBid, caspase-8, -3, and upregulated the phosphorylation of p38 and JNK, ultimately inducing apoptosis in HCT116 and HT29 cells. In addition, this experiment demonstrated that the phosphorylation of p38 MAPK was mediated by ROS.

Oridonin (ORI) is a diterpenoid extracted from *Isodon rubescens* (Hemsl.) H.Hara with strong anti-inflammatory and anti-cancer activities (Li X. et al., 2021). Ren et al. (2016) reported that ORI (0, 5, 10, 15, 20, 25 µM for 24, 48, 72 h) was able to dose- and time-dependently upregulate the levels of BMP7, Bad, and decrease the levels of Bcl-2, promoting apoptosis and arresting the HCT116 cells in the G2 period. Additional study revealed that BMP7 may mediate the antiproliferative effects of ORI in part through activation of p38 MAPK.

Cryptotanshinone (CTS) is also an active quinone diterpene isolated from many botanical drugs such as *Salvia miltiorrhiza* Bunge with anticancer, anti-inflammatory, and immunomodulatory pharmacological effects (Li H. et al., 2021). Xu et al. (2017) observed that CTS (10 µM) induced cell death by autophagy through the ROS-p38 MAPK-NF-kB signaling pathway in SW620-Ad300 cells, which could be blocked by the ROS inhibitor NAC or p38 inhibitor SB202190.

Andrographolide, the most abundant diterpene lactone in botanical drug *Andrographis paniculata* (Burm.f.) Wall. ex Nees, has been shown to alleviate cardiac dysfunction and reduce cardiac hypertrophy and fibrosis (Tian et al., 2023). Yuan et al. (2018) reported that Andrographolide (5,10,20,50 and 100 µM) dose-dependently inhibited NADPH oxidase activation, downregulated ERK1/2, P38 MAPK activation, and effectively suppressed angiogenesis in HCT116 Cells.

Alisol B 23-acetate (AB23A), a tetracyclic triterpenoid derived from botanical drug *Alisma plantago-aquatica subsp*. orientale (Sam.) Sam, which improves allergic asthma (Nam and Im, 2023). Zhu et al. (2021) observed that AB23A (50 mg/kg/day) significantly reduced the phosphorylation of p38-MAPK, ERK, JNK, and upregulated the expression of IL-10 in AOM/DSS colitis-associated cancer (CAC) mouse model, and interfered with the development of CAC.

The essential oil extract from the aromatic medicinal plants *Thymus algeriensis* Boiss. and Reut contains high levels of terpenoids, which can protect nerves (Rezq et al., 2020). Guesmi et al. (2021) reported TS essential oil (0.5–50 pg/mL) dose-dependently activated the ERK1/2, JNK, p38 MAPK, and upregulated the expression of DR5, SP1, CHOP, promoting HCT116 cells apoptosis.

Ganoderma lucidum triterpenes (GLT), a terpene metabolite with antiviral properties extracted from the botanical drug *Ganoderma lucidum* (Curtis) P. Karst (Ahmad et al., 2021). Thyagarajan et al. (2010) reported that GLT (0–1.0 mg/mL; 0–6 h) dose- and time-dependently inhibited phosphorylation of p38, and upregulated the expression of Beclin-1 and LC-3, inducing autophagy in HT-29 cells. P38 inhibitor SB202190 enhanced the processes.

yuanhuacine (YHL-14), a diterpenoid extracted from botanical drug *Daphne genkwa* Siebold & Zucc, can be used to treat lung and breast cancer (Bailly C. 2022). It was found that YHL-14 (2–16 µM) activated p38 MAPK, and upregulated p21 mRNA expression, inducing cell arrest in G2/M phase in HCT116 cells in a dose-dependent manner. (Zhang et al., 2014).

Crocetin, a water-soluble carotenoid which has neuroprotective properties and extracted from botanical drugs such as *Crocus sativus* L (Liu Z. et al., 2023). Khajeh et al. (2020) reported that Crocetin (400, 600, and 800 µM) dose-dependently increased the p-p38 MAPK, downregulated the expression of VEGF and MMP-9 mRNA in HCT-116 cells, thereby inhibiting cell proliferation, migration and angiogenesis. When the concentration of crocetin is 400, 600, 800 μM, the ratio of p-p38/p38 is 1.9, 2.2, 2.5 respectively.

Deguelin, a natural carotenoid extracted from *Mundulea sericea* (Willd.) A.Chev, was used to treat lung cancer (Gao et al., 2020). Chen et al. (2018) observed that Deguelin (0–200 μM for24, 48 h) dose-and time-dependently induced apoptosis in SW620 and RKO cells by activating the p38 MAPK, upregulating cleaved caspase-3, cleaved PARP, downregulating Bcl-2, survivin. P38 MAPK inhibitor SB203580 can block proliferation inhibition. Vivo experiments also showed that Deguelin has an inhibitory effect on CRC.

The above findings suggest that terpenoids play an anti-proliferative, anti-CRC role by mediating MAPK activation of oxidative stress, causing cell cycle arrest, inhibiting angiogenesis, and inducing apoptosis and autophagy.

### 4.3 Saponins

Saponins are plant secondary metabolites with great structural and functional diversity, and are categorized into triterpene saponins and steroidal saponins according to the structure of the glycosides. Natural saponins in herbal medicine have anti-inflammatory, antitumor, antioxidant, and antiviral effects (Zheng Z. et al., 2022). Therefore, the potential of saponins to inhibit CRC is worth investigating.

Ginsenoside Rh1 (Rh1), a tetracyclic triterpenoid saponin extracted from the botanical drug *Panax ginseng* C.A.Mey, is used to treat asthma and allergic inflammation (Jin et al., 2023). Lyu et al. (2019) reported that Rh1 (0, 50,100 µM) was able to suppress the migration and invasion of SW620 cells by decreasing the ratios of p-P38/P38, p-ERK1/2/ERK1-2 and p-JNK/JNK, downregulating the mRNA expression of MMP1 and MMP3, and upregulating the mRNA expression of TIMP3. The ratios of p-38/p38, p-JNK/JNK, p-ERK/ERK were 0.3, 0.2, and 0.6 respectively. *In vitro* experiments demonstrated that oral administration of Rh1 (20 mg/kg) significantly reduced CRC tumor weight.

Rhizoma Panacis Majoris (RPMTG), is a total saponin extracted from *Panax bipinnatifidus var*. bipinnatifidus that can be used in the treatment of rheumatoid arthritis (Ren et al., 2023). Chang et al. (2021) found that RPMTG (0, 100, 200, 250 μg/mL) was able to dose-dependently increase the rate of p-JNK/JNK, p-p38/p38 and decrease p-ERK/ERK, inducing apoptosis and blocking cell cycle in G0/G1 and S phase in HCT116 and SW620 cells, respectively. The above effects were reversed with the addition of JNK inhibitor SP600125 and p38 inhibitor SB203580. In HCT116 cells, at RPMTG 100 and 250 µg/mL, the relative protein levels of p-p38/p38 are about 1.4 and 2.5; in SW620 cells, about 1.1 and 2.

Dioscin, a naturally occurring steroidal saponin found in botanical drugs such as *Dioscorea panthaica* Prain & Burkill, has been shown to reduce oxidative stress and inflammation to minimize intestinal toxicity of chemotherapeutic drugs (Jin S. et al., 2022). Li et al. (2018) reported that Dioscin (0, 1.25, 2.5 and 5 μg/mL for 0, 6, 12 h) time- and dose-dependently inhibited proliferation and promoted apoptosis in RKO, HT-29 cells, which may be related to the activation of JNK/p38 MAPK mediated by ROS. Vivo experiments confirmed that (Tong et al., 2014) Dioscin (30, 60 mg/kg) decreased blood vessel density and inhibited tumor growth in C26 mouse tumor model. Additional study showed that Dioscin downregulated the expression of VEGF, VEGFR2, FAK, Src, and p-ERK1/2, suggesting that Dioscin inhibits angiogenesis by regulating VEGF and MAPK.

Platycodin D (PD) is a triterpenoid saponin extracted from the botanical drug *Platycodon grandiflorus* (Jacq.) A.DC, which has antioxidant and cardiomyocyte protective effects (Wang et al., 2018). Han et al. (2024) found that PD (0, 10, 20 µM) significantly increased the level of p-p38 and upregulated the expression of Bax, cleaved PARP, inducing apoptosis in HT-29 cells in a dose-dependent manner. When the concentrations of Platycodin D were 0, 10, and 20 μM, the ratios of p-ERK/ERK, p-p38/p38, and p-JNK/JNK were approximately1, 0.6, 0.2; 1, 2, 3.5; and 1, 3, 4.5, respectively.

Paris saponin VII, a steroidal saponin from the botanical drug *Trillium tschonoskii* Maxim, which can reverse chemotherapy resistance in breast cancer (Li et al., 2019). Li et al. (2014) reported that Paris saponin VII (0.5, 1.0, 1.5, 2, 2.5, 5 µM for 24, 48 h) was effective in the treatment of HT-29 and SW-620 cells by inhibiting the Ras/MEK1/2/ERK1/2 pathway, increasing cleaved caspase-3-9, PARP, Bax, downregulating Bcl-2, and blocking the cell sycle in G1 phase.

Gynostemma pentaphyllum saponins (GpS), a triterpenoid saponin derived from the botanical drug *Gynostemma pentaphyllum* (Thunb.) Makino, can be used to regulate triglyceride and cholesterol (Xie et al., 2024). Study showed that GpS (500, 750 mg/kg) was able to dose-dependently upregulated the expression of Prdx1 and Prdx2, and downregulate the expression of Raf-1, which acted as an anticancer agent by inhibiting the RAS/RAF/MEK/ERK signaling pathway in ApcMin/+ CRC mice (Tai et al., 2016).

The above findings indicate that saponins are potential natural products to inhibit tumor development *in vitro* and *in vivo* by affecting MAPK, inhibiting angiogenesis, enhancing the efficacy of chemotherapeutic agents, and inducing apoptosis.

### 4.4 Polyphenols

Polyphenols are secondary metabolites, mainly of plant origin, and current research on polyphenols has focused on antioxidant, anti-inflammatory, and anti-tumor properties with a wide range of therapeutic benefits (Bruno and Ghiadoni, 2018; Roszkowski, 2023). Metabolites such as resveratrol, astragalus, and curcumin have been shown to modulate MAPK-induced CRC cell cycle arrest, DNA damage, and apoptosis, and inhibit proliferation and migration.

Curcumin, a natural polyphenol extracted from the botanical drug *Curcuma longa* L, which has anti-inflammatory, anti-cancer effects (Sadeghi et al., 2023). Li et al. (2020) reported that Curcumin (0.10 µM) was able to downregulate the phosphorylation levels of STAT5 and P38, and decrease the expression of HPSE, effectively inhibiting migration and invasion in CT26 cells.

[6]-Gingerol, a polyphenolic metabolite extracted from the botanical drug *Zingiber officinale* Roscoe, which can fight against brain damage (Liu et al., 2020). Radhakrishnan et al. (2014) reported that [6]-Gingerol (5–300 µM for 72 h) induced apoptosis in SW-480 cells through inhibition of ERK1/2 and JNK, activation of caspase-3-7, and upregulation of cleaved PARP.

The above findings suggest that polyphenols regulate MAPK expression, inhibit cell migration and invasion, induce apoptosis, and inhibit the process of tumor metastasis.

### 4.5 Alkaloids

Alkaloids are nitrogen-containing alkaline organic metabolites, mainly from plants, animals and fungi, which exhibit a wide range of biological activities: antitumor, antiviral, antimicrobial, anti-inflammatory (Mondal et al., 2019; Abookleesh et al., 2022). The main mechanisms of action of alkaloids include disrupting angiogenesis, blocking EMT, inhibiting cell proliferation, and inducing apoptosis, which have significant therapeutic effects on CRC.

Lycorine, an isoquinoline alkaloid from botanical drug *Lycoris radiata* (L'Hér.) with anti-inflammatory, anticancer, and antiviral effects (Di Sotto et al., 2023). Gao et al. (2021) revealed that Lycorine (0, 5, 10, 20 µM) increased the level of E-calmodulin, and decreased the expression of N-calmodulin, β-linker, waveform protein, Snai1 protein, blocking the EMT process, and inhibiting metastasis and invasion in CT-26, HCT116, and HT-29 cells, which related to the inhibition of MAPK.

Piperlongumine (PPLGM) is an amide alkaloid isolated from *Piper longum* L and serves effectively in anti-proliferative and anti-inflammatory (Thatikonda et al., 2020). Study (Randhawa et al., 2013) showed that PPLGM (0, 10 and 20 µM for 0, 5, 10, 15, 30 min) was able to upregulate the levels of ROS, and activate MEK/ERK pathway, inducing apoptosis in HT-29 cells as manifested by apoptotic vesiculation, chromatin condensation and caspase-3 cleavage.

Matrine (Mat), a tetracyclic quinolizine alkaloid extracted from botanical drug *Sophora flavescens* Aiton, has neuroprotective effects (Chhabra and Mehan, 2023). Du et al. (2023) found that Mat (0, 0.25, 0.5, 1 µM for 24, 48 h) was able to time- and dose-dependently downregulate the phosphorylation levels of JNK and ERK, to decrease the expression of the Cldn9 gene, to inhibit the formation of VMs, to downregulate N-calmodulin, MMP2, MMP9, resulting in the inhibition of cell proliferation, migration and invasion in CT26 cells.

Homoharringtonine (HHT), a natural alkaloid derived from the *Cephalotaxus hainanensis* H.L.Li, can be used to treat breast cancer (Wang L. B. et al., 2021). Shi et al. (2020) reported that HHT (0.1, 0.2, 0.4, 1.0 µM for 2, 6, 24, 48 h) dose- and time-dependently inhibited EphB4, and downregulated phosphorylation of MEK, ERK1/2, and decreased the expression of Bcl-2, Mcl-1 to promote apoptosis in LoVo cells. Animal experiments showed that HHT (1 mg/kg) inhibited tumor growth in male BALB/C nude mouse.

Tetrandrine is a isoquinoline alkaloid extracted from the botanical drug *Stephania tetrandra* S.Moore with good antibacterial, anti-inflammatory, and anticancer effects (Bhagya and Chandrashekar, 2022). Wu et al. (2010) found that Tetrandrine induced apoptosis by activating P38 MAPK in CT-26 cells.

The above findings suggest that alkaloids can inhibit the EMT process in CRC cells, inhibit invasion and metastasis, block angiogenesis, induce apoptosis, and inhibit tumor growth.

### 4.6 Other metabolites

Vanillin, a 4 hydroxy 3 methoxybenzaldehyde extracted from the plant *Vanilla pompona* Schiede, could be used to treat inflammation and regenerate intervertebral disc (Zhu et al., 2023b). Li G. et al (2021) found Vanillin (0, 2.5, 3.5 for 48 h) dose-dependently activated ASK1-p38 MAPK pathway and downregulated the expression of NNMT, inducing apoptosis in HT-29 and SW480 cells. *In vivo* experiment showed that, compared with the use of 5-FU alone, the combination of vanillin with 5 Fu has an excellent synergistic effect which could more significantly induce apoptosis and inhibit tumor growth.

Podophyllotoxin (PT), an aryl tetrahydronaphthalene lignan with antiviral effect extracted from the botanical drugs such as *Podophyllum versipelle* Hance (Cui et al., 2020). Lee et al. (2021) demonstrated that PT (0.1, 0.2, 0.3 µM for 24, 48 h) could dose-dependently induce apoptosis and block the cell cycle at G2/M phase in HCT116 cells by increasing the level of ROS, increasing the phosphorylation of p38 MAPK and upregulating ER stress markers such as RP78, CHOP.

Ursolic acid, a pentacyclic triterpene acid, extracted from botanical drugs such as *Scleromitrion diffusum* (Willd.) R.J.Wang and *Prunella vulgaris* L, was used to prevent myocardial ischemic disease (Xu L. L. et al., 2023). Shan et al. (2016) found that Ursolic acid (15, 30, 60 μmol/L) dose-dependently downregulated p-MEK1/2, p-ERK1/2, p-p38, p-JNK, and increased cleaved caspase-3, -9, -8, inducing apoptosis in SW620 and RKO cells. What’s more, these effects significantly increased when Ursolic acid is used in combination with oxaliplatin.

Di Yu (DY), derived from the botanical drug *Sanguisorba officinalis* L with inhibitory effects on non-small cell lung cancer (Li H. et al., 2022). Zhang et al. (2023) found that 100 μg/mL DY combined with 100 µM 5-FU significantly downregulated the Ras/MEK/ERK pathway, and increased the expression of Bax, cleaved caspase-3, -9, cleaved PARP, enhancing the chemosensitivity to 5-FU, and inhibiting the migration of drug-resistant cells in RKO-R and HCT15-R cells.

Ferulic acid (FA), a phytophenolic acid found in botanical drug such as *Angelica sinensis* (Oliv.) Diels, has anti-inflammatory, antioxidant properties (Ghasemi-Dehnoo et al., 2023). Chen et al. (2023) reported that FA (20, 40, 80 mg/mL·kg-1 BW for 20 days) could dose-dependently increase the phosphorylation of JNK and ERK, and significantly upregulate the expression of BAX, meanwhile downregulate BCL-2 protein levels in CT26 cell-induced CRC mice. The results indicated that FA inhibit CT26 cell viability and promote apoptosis by upregulating the activities of JNK and ERK.

8-Gingerol is a natural metabolite extracted from *Zingiber officinale* Roscoe that can inhibit excessive autophagy and apoptosis in cardiomyocytes (Xue et al., 2021). It was found that 8-Gingerol (0, 30, 70, and 100 µM) dose-dependently inhibited the EGFR/STAT/ERK pathway and downregulated cyclin D1, c-Myc, and MMP2 expression in HCT116 and DLD1 cells, as well as induced cell cycle arrest in the G0/G1 phase and inhibited cell proliferation and migration. In addition, 8-gingerol can reduce the effective concentration of 5-Fu, thus reducing the toxicity of 5-Fu in drug combination therapy (Hu et al., 2020).

Alantolactone (ALT), a sesquiterpene lactone extracted from *Inula helenium* L, which can be used to fight against liver cancer, lung cancer (Cai et al., 2021). Cao et al. (2019) found that ALT (10 µM) combination with oxaliplatin (40–120 µM) significantly increased ROS level, and upregulated the phosphorylation of JNK and p38 MAPK, inhibiting cell proliferation as well as inducing cell apoptosis in HCT116 and RKO cells. JNK inhibitor SP600125 or p38 inhibitor BMS-582949 could partially reverse the anti-tumor effect.

Curcumol, a guaiacoloid sesquiterpenoid semiketone extracted from the botanical drug *Curcuma longa* L, can be used to alleviate liver fibrosis (Zheng Y. et al., 2022). Wang et al. (2015) reported that Curcumol (0.05, 0.1, 0.2, 0.4 µM/mL for 24, 48, 72, 96, 120 h) time- and dose-dependently decreased the protein level of IGF-1R, and upregulated the expression of phosphorylated p38, inducing apoptosis in LoVo cells. *In vivo* experiments demonstrated that curcuminol inhibited tumor development.

Salidroside, a glycoside derived from the botanical drug *Rhodiola rosea* L, may improve ameliorate Alzheimer’s disease (Cai et al., 2022). EL-kott et al. (2021) reported that Salidroside showed to be effective in the treatment of HT29 cells by activating protein kinase R, increasing the levels of p-JNK, p38 MAPK, upregulating caspase-3, -8 and inducing apoptosis.

Sulforaphane (SFN), a naturally occurring isothiocyanate widely found in *Brassica oleracea* L, fights against with gastric cancer (Li S. et al., 2022). Sah et al. (2024) found that SFN (1, 2, 5, 10 µM) dose-dependently reduced ROS and the expression of MAPK, while decreasing the expression of IL-1β, IL-6 and resulting in inhibition of cell proliferation and invasiveness in HT-29 cells.

Imperatorin, a furanocoumarin extracted from the botanical drug *Angelica dahurica* (Hoffm.) Benth. and Hook.f. ex Franch. and Sav which can inhibite osteosarcoma (Lv et al., 2021). Mi et al. (2017) revealed that Imperatorin (50, 100, 150 µM) dose-dependently inhibited the phosphorylation of ERK1/2, JNK, p38, disrupted HIF-1α protein synthesis, and downregulated the expression of VEGF and CD31, inhibiting cell proliferation and angiogenesis, and blocking the cell cycle at G1 phase in HCT116 cells.

Ganoderma lucidum polysaccharide (GLP), a water-soluble polysaccharide extracted from the botanical drug *Ganoderma lucidum* (Curtis) P. Karst, which has antioxidant, anticancer, and neuroprotective effects (Liu X. et al., 2023). Pan. et al. (2019) found that GLP (0, 2.5, 5, 10 mg/mL for 24, 48, 72 h) phosphorylated MAPK/ERK in a dose-dependent manner, and induced the expression of LC3-II, p62, which decreased cellular autophagic flux and blocked autophagosome-lysosome fusion, inducing apoptosis in HT-29 and HCT116 cells. The above effects were reserved by MAPK/ERK inhibitor PD98059.Animal experiments showed that 300 mg/kg of ALP inhibited tumor growth and autophagic flow in xenografts.

Many experiments have limitations such as short cycles, lack of control groups, and absence of pharmacological toxicity tests. However, considering the limited investment in this field at present, we are lenient about these issues.

**FIGURE 2:**
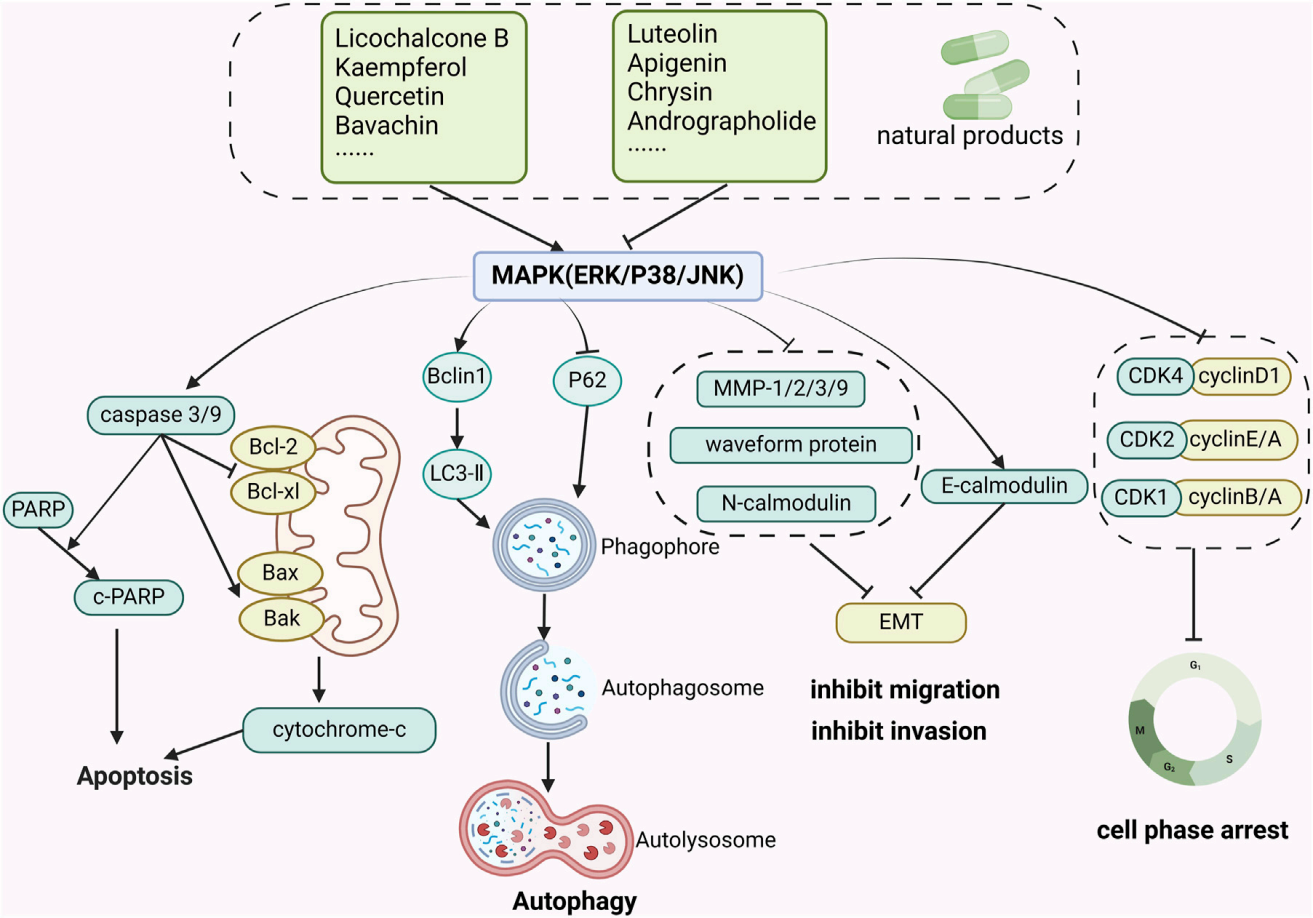
The mechanism of natural products modulating MAPK for CRC. Created with Biorender.com.

In a nut, a large body of evidence suggested that botanical drug extracts (e.g., flavonoids, terpenoids, saponins, polyphenols, alkaloids) could modulate the MAPK pathway, which demonstrated excellent anti-CRC capabilities. The review described mechanisms of natural metabolites in Figure 2.

## 5 Conclusion

In this review, we summarized the role of the MAPK pathway in the development of CRC from cell growth and proliferation, invasion and migration, apoptosis, autophagy, oxidative stress, pathologic angiogenesis, and chemotherapy resistance, suggesting that the MAPK pathway may be a potential therapeutic target for CRC. We similarly discuss the preventive and therapeutic effects of natural products on CRC by modulating the MAPK pathway. Natural products are characterized by multi-target and multi-pathway collaboration, mainly including flavonoids, terpenoids, saponins, polyphenols, and alkaloids, etc., and they exhibit adequate anti-metastatic and anti-tumor effects mainly by inhibiting CRC various ways.

At present, the use of natural products to treat diseases has become a trend. Research shows that many natural products have excellent effects due to high safety, multiple targets, rich variety, low toxic and side effects, which have great application potential in the increasingly common diseases. What’s more, natural products can inhibit the drug resistance of tumor cells to 5-Fu and OXR. The combination can enhance the drug effects, improve the effectiveness of chemotherapy, alleviate adverse reactions, and overcome drug resistance. However, considering the difficulties in extracting natural products from botanical drugs and their low bioavailability, studies are currently limited to the scope of preclinical experiments, with few studies on CRC patients and a lack of evidence based on high-quality clinical findings. On the one hand, the metabolites of natural products are diverse and often work synergistically, which blocks precise treatment and drug development. On the other hand, considering the requirements of regulations, ethics, the guarantee of safety, and the high research costs, there are currently few clinical trial data. Therefore, we can optimize the research technical routes and methods to quickly and accurately analyze the metabolites and action targets of natural products. At the same time, more animal experiments should be conducted to further study the metabolic process of drug extracts *in vivo*. In addition, potential upstream mechanisms and possible toxicity of natural products need more elucidation.

In conclusion, we reviewed that herbal natural products can prevent and treat CRC by modulating the MAPK signaling pathway, which provides a theoretical basis and new ideas for further exploration of the mechanism of natural metabolites. In order to fully elucidate the preventive and therapeutic effects of natural products on CRC, further studies are needed to illustrate the specific mechanisms involved.

## Glossary

CRC: Colorectal cancer
MAPK: Mitogen-activated protein kinase
AS-IV: Astragaloside IV
HA: Holothurin A
ERK: extracellular regulating kinase
p38 MAPK: p38 mitogen-activated protein kinase
JNK: Jun N-terminal kinase
RAS: Ras small GTPase
EGFR: epidermal factor receptor
PAMPs: Pathogen-Associated Molecular Patterns
DAMPs: Danger-Associated Molecular Patterns
eEF1A1: Eukaryotic elongation factor 1 A1
ROS: reactive oxygen species
MIF: migration inhibitory factor
ILT4: Immunoglobulin-like transcript 4
LCB: Licochalcone B
Bcl-2: B-cell lymphoma-2
LC3II: light chain 3 II
LC3I: light chain 3 I
Ech: Echinatin
OMEO: Origanum majoana Essential Oil
Tan I: Tanshinone I
ORI: Oridonin
CTS: Cryptotanshinone
AB23A: Alisol B 23-acetate
GLT: Ganoderma lucidum triterpenes
RPMTG: Rhizoma Panacis Majoris
PD: Platycodin D
GpS: Gynostemma pentaphyllum saponins
PPLGM: Piperlongumine
FA: Ferulic acid
GLP: Ganoderma lucidum polysaccharide
TNF-α: tumor necrosis factor-α
NF-κB: Nuclear factor kappa-β
